# Higher body‐mass index modifies time‐resolved maternal autonomic cardiac–uterine coupling during the first stage of human labour

**DOI:** 10.1113/EP093339

**Published:** 2026-04-25

**Authors:** Carlos Gabriel Varela‐Albarrán, José Javier Reyes‐Lagos, Laura Mercedes Santiago‐Fuentes, Guadalupe Dorantes‐Méndez, Eric Alonso Abarca‐Castro, Paula Romina Soria, Araceli Espinosa‐Guerrero

**Affiliations:** ^1^ School of Medicine Autonomous University of the State of Mexico (UAEMéx) Toluca de Lerdo Mexico; ^2^ Bioelectronics Section Electrical Engineering Department Center for Research and Advanced Studies Mexico City Mexico; ^3^ School of Sciences Autonomous University of San Luis Potosí (UASLP) San Luis Potosí Mexico; ^4^ Division of Biological and Health Sciences Autonomous Metropolitan University Lerma Campus (UAM‐L) Lerma de Villada Mexico; ^5^ Faculty of Engineering, Institute of Biomedical Engineering University of Buenos Aires Buenos Aires Argentina; ^6^ Argentine Institute of Mathematics ‘Alberto P. Calderón’ CONICET Buenos Aires Argentina; ^7^ Gynecology and Obstetrics Hospital Maternal and Child Institute of the State of Mexico (IMIEM) Toluca de Lerdo Mexico

**Keywords:** autonomic–uterine coupling, electrohysterography, maternal HRV, obesity in pregnancy, phase synchronization, SampEn

## Abstract

We investigated whether higher body mass index (BMI) modifies time‐resolved maternal autonomic control and maternal cardio‐electrohysterographic coupling (MCEC) during the first stage of labour. Seventy‐nine women were studied and, for the present analysis, grouped as Control (C; *n* = 41) and high BMI (HBMI; *n* = 38). Maternal inter‐beat intervals were used to derive time‐resolved cardiac sympathetic (CSI) and cardiac vagal (CVI) indices, while uterine activity was obtained from electrohysterography. Compared with controls, HBMI showed higher median CSI and CVI and higher uterine irregularity, consistent with overall autonomic overactivation (increased sympathetic and vagal‐related modulation) as captured by these indices. Phase‐based coupling analyses indicated a shift in vagal‐related phase timing that correlated with BMI as a continuous variable, suggesting that altered MCEC was primarily expressed as a shift in vagal–uterine phase timing. Overall, higher BMI was associated with altered maternal autonomic dynamics and vagal–uterine timing during labour. These findings support BMI‐aware, non‐invasive intrapartum monitoring as a potential approach to improve physiological interpretation and help guide more individualized labour management.

## Abstract (highlights)

- **What is the central question of this study?**
  How does body mass index (BMI) affect time‐resolved maternal autonomic dynamics and their phase‐based coupling to uterine activity during the first stage of labour?
- **What is the main finding and its importance?**
  Higher BMI showed greater cardiac sympathetic index and cardiac vagal index central tendency (sympathetic and vagal overactivation) and a BMI‐related vagal–uterine phase offset (Δφ), whereas phase‐locking strength (λ) was largely unchanged. Thus, BMI mainly shifts autonomic modulation and vagal–uterine timing, supporting BMI‐aware interpretation of non‐invasive intrapartum autonomic–uterine monitoring.

## 1 INTRODUCTION

By the end of 2024, Mexico's estimated maternal mortality ratio (MMR) was 26.1 deaths per 100,000 estimated births, with hypertensive disease, obstetric haemorrhage, pregnancy complications and abortion among the leading causes (Gobierno de México, 2024). The most recent weekly report on immediate maternal death notification for 2025 indicated a comparable MMR of 25.9, underscoring the persistence of this public‐health challenge (Gobierno de México, 2025).

Maternal overweight and obesity before conception and excessive gestational weight gain are well‐established risk factors for complications that heighten maternal and perinatal morbidity and mortality, including gestational hypertension and preeclampsia, future cardiovascular disease, gestational diabetes, preterm birth, anaesthetic complications, operative delivery, intrapartum haemorrhage, thromboembolic events and depression (Mierzyński et al., 2023). Globally, the burden is rising; in 2005, 23.2% of adults were overweight and 9.8% were obese. Recent forecasts suggest that by 2050, the number of adults living with overweight and obesity could reach 3.80 billion, representing more than half of the projected global adult population (Ng et al., 2025). Echoing this trend, a Mexican study in women with high‐risk pregnancy reported that 75% were overweight or obese at the beginning of pregnancy (Hernández‐Higareda et al., 2017). The implications reach beyond the mother: maternal obesity is associated with placental metabolic, inflammatory and epigenetic alterations that may mediate adverse offspring outcomes (Louwen et al., 2024).

Obesity is accompanied by alterations in the autonomic nervous system (ANS) function that may modulate labour physiology. Hyperinsulinaemia and adipokine–CNS cross‐talk are associated with heightened sympathetic outflow, faster heart rate and reduced heart rate variability (HRV), findings supported by haemodynamic and autonomic responses to insulin in lean versus obese individuals (Muscelli et al., 1998; Russo et al., 2021). Obesity is also associated with depressed parasympathetic activity and impaired cardiac autonomic control (Tonhajzerova et al., 2025; Yadav et al., 2017). HRV offers a non‐invasive window onto autonomic modulation, with measurement and interpretation standardized by consensus guidelines (Task Force, 1996). During labour, the autonomic state, and thus maternal cardiovascular control, may be further influenced by the mode of delivery (Papadopoulos et al., 2021), the emotional state of the mother (Pinna & Edwards, 2020), the expectation about the birth of her child (Webb et al., 2021), parity‐related factors (noted across mammalian labour models) (Felici et al., 2023), and pharmacological augmentation such as oxytocin, which increases autonomic cardiac control (Norman et al., 2011).

Because HRV can be obtained non‐invasively from the maternal electrocardiogram (ECG), it is an attractive candidate biomarker for indirect assessment of sympathetic–parasympathetic dynamics during labour. Conventional HRV analysis relies on time‐ and frequency‐domain metrics—for example, standard deviation of NN intervals (SDNN), root mean square of successive differences (RMSSD), number of pairs of successive NN intervals > 50 ms (NN50), percentage of successive NN intervals > 50 ms (pNN50), high‐frequency power (HF 0.15–0.40 Hz), low frequency‐power (LF; 0.04–0.15 Hz), and the LF/HF ratio—originally framed within spectral analysis of autonomic function and subsequently harmonized by standards documents (Pomeranz et al., 1985; Task Force, 1996; Yadav et al., 2017). While HF power largely reflects vagal activity, the LF band reflects a mixture of sympathetic and parasympathetic influences, complicating interpretation in dynamic physiological contexts (Pomeranz et al., 1985; Task Force, 1996; Yadav et al., 2017). Crucially, classical HRV metrics presume (approximately) stationary segments, a condition that may often be violated during human labour, where uterine activity and maternal cardiovascular responses evolve rapidly and non‐linearly over time. Thus, a time‐resolved perspective is warranted to capture the evolving autonomic and uterine processes during labour.

To address these limitations, Candia‐Rivera et al. proposed robust, time‐resolved indices of autonomic outflow derived from the Poincaré distribution of successive inter‐beat intervals (IBI): the cardiac sympathetic index (CSI) and the cardiac vagal index (CVI). These indices leverage ellipse geometry to provide interpretable proxies of sympathetic (long‐axis) and parasympathetic (short‐axis) dynamics without strict stationarity assumptions (Candia‐Rivera et al., 2025b). Building on this perspective, we define maternal cardio‐electrohysterographic coupling (MCEC) as the two‐way interaction between uterine electrical/contractile activity (electrohysterogram‐ or TOCO‐like signals) and maternal HRV. To the best of our knowledge, neither CSI/CVI nor MCEC has been evaluated between normal‐weight and high body mass index (BMI) groups during the first stage of labour, despite the biologically plausible impact of excess adiposity on autonomic and uterine dynamics.

Using this novel methodology based on time‐resolved CSI and CVI, we sought to characterize dynamic autonomic activity in normal‐weight versus high BMI parturients, and to quantify MCEC between groups during the first stage of labour; additionally, we assessed associations with BMI as a continuous variable. We hypothesized that higher BMI would be associated with altered maternal autonomic dynamics (CSI/CVI) and modified MCEC, expressed as shifts in phase timing and/or changes in phase‐locking properties, with potential implications for clinical decision‐making in the management of pregnancy and labour.

## 2 METHODS

### 2.1 Ethical approval

This study was approved by the Education, Research, Training and Ethics Subcommittee of the Gynecology and Obstetrics Hospital, Maternal and Child Institute of the State of Mexico (IMIEM), Toluca, Mexico (Protocol ID: 300/092/2022). Written informed consent was obtained from all participants prior to any recording. The study conformed to the standards set by the *Declaration of Helsinki*, except for registration in a database.

### 2.2 Data acquisition and signal processing: Maternal RR intervals and EHG‐derived uterine activity

We enrolled 79 parturient women in the third trimester of gestation, all in the first stage of labour, who were receiving care in the Labor and Delivery unit of the Gynecology and Obstetrics Hospital at the Maternal and Child Institute of the State of Mexico (IMIEM), Toluca de Lerdo, Mexico. Participants were stratified by current BMI at labour admission, according to WHO definitions (WHO, 2016): normal weight (18.5–24.9 kg/m^2^), overweight (25.0–29.9 kg/m^2^), and obesity (≥30.0 kg/m^2^). For the present analysis, the overweight and obesity categories were merged into a single high BMI group (HBMI) to improve interpretability and robustness, yielding two groups: normal weight (C; *n* = 41) and high BMI (HBMI; *n* = 38). All participants received a clinical diagnosis of labour, characterized by the presence of uterine activity and cervical effacement; this diagnosis was made by hospital physicians. In addition, maternal clinical parameters were recorded during labour, including systolic and diastolic blood pressure and body temperature.

**FIGURE 1:**
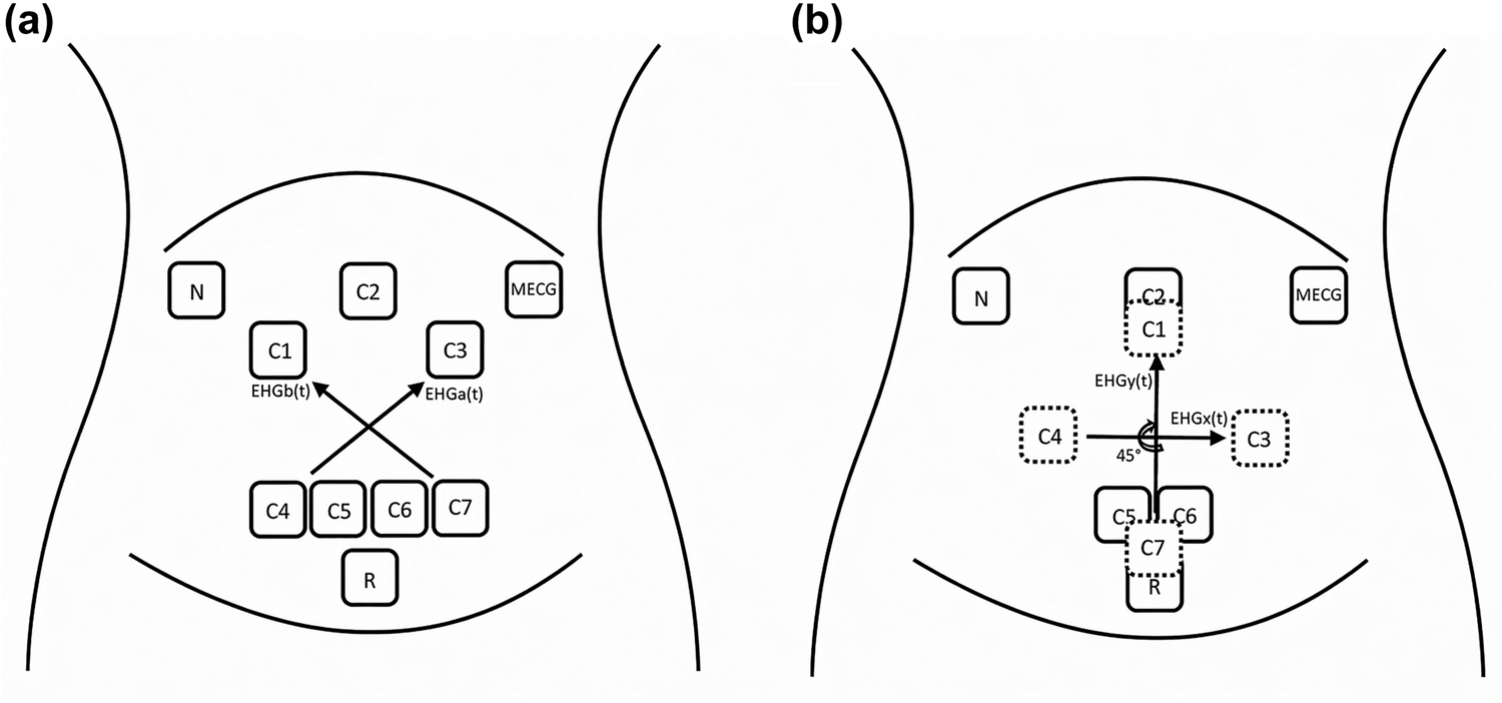
Electrode montage and derivation of EHG components. (a) Transabdominal monopolar electrode arrangement used with the Babycard maternal–fetal monitor. Corner electrodes form two diagonal bipolar derivations, EHG_a_ = C3 − C4 and EHG_b_ = C1 − C7. Additional electrodes indicate the neutral (N), reference/ground (R), and maternal ECG input (MECG). (b) A 45° clockwise rotation of the {EHG_a_, EHG_b_} basis aligns the projections with the horizontal (*X*) and vertical (*Y*) axes, yielding the orthogonal components EHG*_x_* and EHG*_y_* for uterine activity analysis.

Continuous transabdominal recordings (21 min) were acquired using the Babycard maternal–fetal monitor (Scientific and Research Centre ‘Kahl Medica’, Kharkiv, Ukraine) (Viunytskyi & Shulgin, 2017a,b) with a sampling frequency of 1000 Hz using the electrode montage shown in Figure 1a. Maternal RR or interbeat interval (IBI) time series were derived from R‐peaks detected in the maternal ECG using CardioLab software (Scientific and Research Centre ‘Kahl Medica’, Kharkiv, Ukraine) and processed with artifact‐adaptive filtering to remove ectopic or erroneous R–R intervals prior to analysis (Wessel et al., 2000).

Uterine activity was derived from the electrohysterogram (EHG) as follows. Two diagonal bipolar derivations were formed from the abdominal array by subtracting the monopolar signals of electrode pairs 3–4 and 1–7 to obtain EHG_a_ and EHG_b_ (Figure 1a). These were then rotated by 45 degrees to yield two orthogonal components, EHG*_x_* or *X* (horizontal) and EHG*_y_* or *Y* (vertical), approximating longitudinal and transverse propagation (Figure 1b), consistent with previous studies (Albaladejo‐Belmonte et al., 2022; Reyes‐Lagos et al., 2024). Each component was band‐pass filtered between 0.2 and 1.0 Hz using a zero‐phase fourth‐order Butterworth filter. To minimize start‐up artefacts, the first minute of each recording was discarded, after which the signals were resampled to 20 Hz. A root mean square (RMS) envelope was computed with a 20‐s window and smoothed with a 30‐s moving average to emphasize slow contractile dynamics or TOCO‐like signals. An adaptive baseline and a detection line were estimated over 4‐min windows, with a step size of 1 min, using the modal amplitude as the baseline and the baseline plus 25% of the local range as the detection line.

### 2.3 Estimation of cardiac sympathetic and parasympathetic indices (CSI and CVI)

**FIGURE 2:**
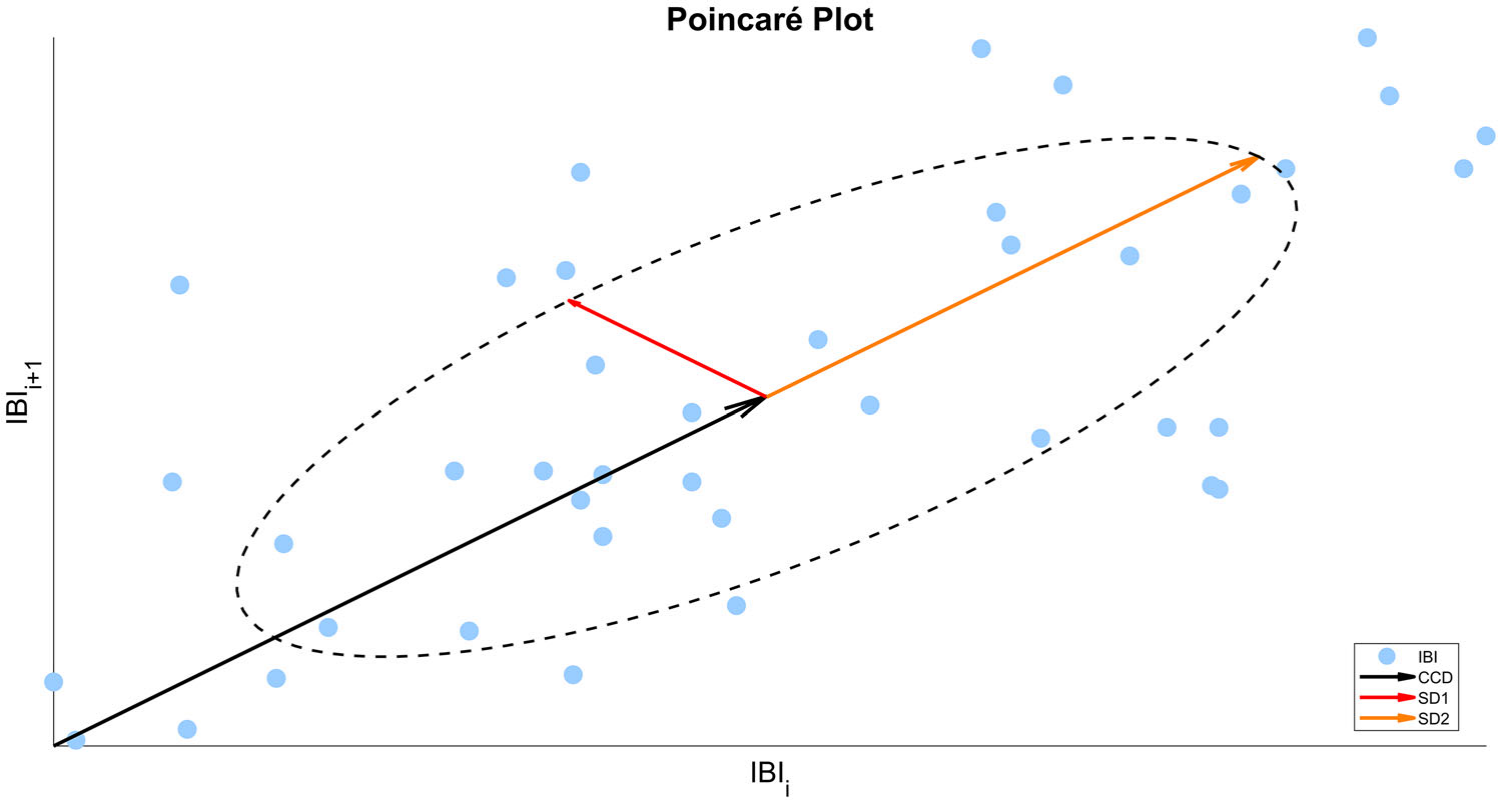
Poincaré map illustrating the sequential changes in the IBIs, the minor (SD_01_) and major (SD_02_) ratios of the formed ellipse, and the distance from the origin to the centre of the ellipse (CCD).

To obtain the cardiac sympathetic and parasympathetic/vagal indices (CSI and CVI), we followed the methodology described by Candia‐Rivera et al. (2025b). The first minute of the maternal RR‐interval series was excluded to remove start‐up artifacts, and the remaining signal was divided into non‐overlapping windows of 15 s. For each window, a Poincaré plot was constructed by mapping successive inter‐beat intervals (IBI*_i_* vs. IBI*_i_*_+1_). The distribution typically adopts an elliptical shape, from which three parameters were derived: SD1, the minor semi‐axis, representing short‐term heart rate fluctuations; SD2, the major semi‐axis, associated with long‐term fluctuations; and CCD, the cardiac cycle duration, defined as the distance from the origin to the ellipse centroid. The time‐resolved values were computed as follows:

(1) $$\mathrm{CCD}\;\left(t\right)=\sqrt{\mathrm{mean}{\left(\mathrm{IB}I_{i,\text{…},n-1}\right)}^{2}+\mathrm{mean}{\left(\mathrm{IB}I_{i+1,\text{…},n}\right)}^{2}}\;$$

(2) $${\mathrm{SD}}_{1}\;\left(t\right)=\sqrt{\lambda_{\Omega_{t}}\left(1,1\right)}=\sqrt{\frac{1}{2}\mathrm{std}{\left(\mathrm{IB}{I^{\prime }}_{\Omega_{t}}\right)}^{2}}\;$$

(3) $$SD_{2}\;\left(t\right)=\sqrt{\lambda_{\Omega_{t}}\left(2,2\right)}=\sqrt{\left|2\mathrm{std}{\left(\mathrm{IB}I_{\Omega_{t}}\right)}^{2}-\frac{1}{2}\mathrm{std}{\left(\mathrm{IB}{I^{\prime }}_{\Omega_{t}}\right)}^{2}\right|}\;$$

where λ_Ω_ *_t_* is the matrix with the eigenvalues of the covariance matrix of IBI*_i_*_,…,_ *_n_*_−1_ and IBI*_i_*_+1,…,_ *_n_*, with Ω*_t_*: *t* – *T* ≤ *t_i_* ≤ *t*, *n* is the length of IBI in the time window Ω*_t_* (where *t* is the time in seconds of the record, *T* is the width of the time window and *t_i_* is the time at which a peak R occurs), IBI′ is the derivative of IBI, std refers to the standard deviation; and ∣·∣ is the absolute value. A schematic representation of the ellipse (SD1, SD2 and CCD) is provided in Figure 2.

Next, time‐resolved indices were integrated to obtain the CVI and cardiac CSI indices, as follows:

(4) $$D\left(t\right)=\bar{\mathrm{CCD}}\;\left(t\right)+\mathrm{CC}D_{0}$$

(5) $$\mathrm{CVI}\left(t\right)=k_{p}\;\left(\bar{SD_{1}}\left(t\right)+SD_{01}\right)+D\left(t\right)$$

(6) $$\mathrm{CSI}\left(t\right)=k_{s}\;\left(\bar{SD_{2}}\left(t\right)+SD_{02}\right)+\tilde{D}\left(t\right)$$

where $\bar{SD_{x}}$ is the demeaned SD*_x_* and $\tilde{D}$ is the flipped *D* with respect to the mean, *k*_p_ and *k*_s_ define the weight of the fast and slow HRV oscillations with respect to changes in the baseline CCD (*k*_p _= 1, *k*_s _= 10 as recommended in Candia‐Rivera et al., [2025b]). CCD_0_, SD_01_, and SD_02_ correspond to the calculation considering the entire record with the purpose of recentring the time‐resolved estimations of CCD, SD_1,_ and SD_2_.

CSI and CVI were normalized to *Z*‐scores before group‐level comparisons. The normalized time series were further segmented using a 5‐min sliding window with a 10‐s step, resulting in 91 windows for the 20 min of analysed data. Within each window, the median, RMS value, kurtosis and skewness were extracted as descriptive features. All analyses were conducted in MATLAB R2024a (MathWorks, Natick, MA, USA) using the publicly available code by Candia‐Rivera (freely available at https://github.com/diegocandiar/robust_hrv).

### 2.4 Phase extraction (Δφ) and phase‐locking (λ) of autonomic indices with uterine contractions

To quantify phase coupling between autonomic indices and uterine contractions in labour, we first made all series equispaced: CSI, CVI and the TOCO‐like envelopes from EHG *X* and *Y* were aligned in time and interpolated to 20 Hz. Within the same analysis windows described previously, each band‐limited signal (𝑡) was converted to its analytic form via the Hilbert transform (Gabor, 1946; Pikovsky et al., 2002): $\vartheta \left(t\right)=x\left(t\right)+i\;\hat{x}\left(t\right)$ with $\hat{x}\left(t\right)=H\left\{x(t)\right\}$; the instantaneous phase was then ϕ(t)=argϑ(t).

For every window, we formed phase‐difference series for the four pairs of interest:

(7) $$\Delta \phi_{\mathrm{CSI},X}\left(t\right),\Delta \phi_{\mathrm{CSI},Y}\left(t\right),\Delta \phi_{\mathrm{CVI},X}\left(t\right),\Delta \phi_{\mathrm{CVI},Y}\left(t\right),$$

wrapping differences to (−π,π]. The phase synchronization index *λ* (Rosenblum et al., 1996) was computed for each pair as:

(8) $$\lambda =\sqrt{\;{\langle \sin\Delta \phi \left(t\right)\rangle }^{2}+{\langle \cos\Delta \phi \left(t\right)\rangle }^{2}\;}\;,$$

The mean phase difference Δϕ(t) and the mean phase synchronization index λ were calculated for all cases—that is, for every CSI/CVI–TOCO (*X*/*Y*) pair within each analysis window and participant between the Control and HBMI groups—yielding window‐level λ values (and corresponding phase‐difference series). Δφ quantifies relative phase timing (lead/lag under our convention), with values closer to 0 indicating more in‐phase timing. λ quantifies phase‐locking strength, with higher values indicating stronger locking.

### 2.5 Assessment of uterine activity irregularity

To quantify irregularity in the TOCO‐like envelopes derived from the EHG's horizontal or *X* and vertical or *Y* components, sample entropy (SampEn) was computed on the complete analysed recording (20 min after discarding the first minute). Prior work in these cohorts has demonstrated differences in uterine dynamics using recurrence analyses of the raw EHG (Reyes‐Lagos et al., 2024); however, irregularity of the TOCO‐like envelopes themselves has not been established. Following the original definition (Richman & Moorman, 2000), SampEn for *X* (SampEn*_X_*) and *Y* (SampEn*_Y_*) were estimated with embedding dimension *m* = 1 and tolerance *r *= 0.2 × SD (computed from the full series) (Sarlabous et al., 2019). SampEn values were obtained separately for *X* and *Y*, and summarized per participant.

### 2.6 Statistical analysis

All analyses were performed using non‐parametric methods (two‐sided, α = 0.05). Group comparisons between C and HBMI were conducted with the Mann–Whitney *U*‐test. Clinical variables were summarized as median [Q1–Q3], whereas categorical variables were summarized as *n* (%) and compared between groups using the chi‐square (*χ* ^2^) test. For time‐resolved analyses, autonomic descriptors and phase‐based coupling measures were computed per participant in 5‐min windows with a 10‐s step. Phase‐based measures included the mean phase difference (Δφ) and the phase synchronization index (λ) for each CSI/CVI–TOCO (*X*/*Y*) pair. For inference, window‐level estimates were summarized per participant as the median across all windows (one value per participant). Figures display individual participant values and group medians with range. Associations with BMI as a continuous variable were assessed using Spearman's rank correlation (ρ). All computations were performed in MATLAB R2024a. Additional statistical analyses and plotting were performed in GraphPad Prism (version 10; GraphPad Software, Boston, MA, USA).

## 3 RESULTS

### 3.1 Clinical characteristics for normal weight and high BMI groups

Table 1 summarizes maternal clinical and obstetric characteristics by group (values as median [Q1–Q3] for continuous variables and *n* (%) for categorical variables). As expected, weight and BMI significantly differed between groups (Mann–Whitney *P* = 2.41 × 10^−^^8^ and *P* = 3.70 × 10^−^^1^ ^3^, respectively). Median weight was 62.5 [59.0–66.6] kg in C and 73.2 [69.5–80.0] kg in HBMI, and median BMI was 21.8 [20.5–22.9] kg/m^2^ in C and 27.6 [25.8–29.0] kg/m^2^ in HBMI. Diastolic pressure was significantly higher in HBMI than in C (79 [70–80] vs. 70 [67–79] mmHg; *P* = 0.020). No significant between‐group differences were observed for age, gestational age, systolic pressure, temperature, cervical dilatation, cervical effacement, contractions per 10 min, labour phase, oxytocin administration/dose or parity (all *P* > 0.05). Epidural block was not administered in either group.

**TABLE 1:** Clinical and obstetric characteristics of participants in the Control (C) and high BMI (HBMI) groups during the first stage of labour.

Variable	C (*n* = 41)	HBMI (*n* = 38)	*P*
Age (years)	22 [20–26]	22 [20–27]	0.976
Weight (kg)	62.5 [59.0–66.6]	73.2 [69.5–80.0]	**2.41 × 10^−8^**
BMI (kg/m^2^)	21.8 [20.5–22.9]	27.6 [25.8–29.0]	**3.70 × 10^−13^**
Gestational age (weeks)	39.0 [38.1–40.0]	39.0 [38.3–40.3]	0.279
Systolic pressure (mmHg)	116 [110–120]	120 [112–125]	0.065
Diastolic pressure (mmHg)	70 [67–79]	79 [70–80]	**0.020**
Temperature (°C)	36.0 [36.0–36.2]	36.0 [36.0–36.5]	0.077
Cervical dilatation (cm)	3 [2–4]	5 [2–5]	0.102
Cervical effacement (%)	60 [50–70]	60 [50–70]	0.394
Contractions (per 10 min)	2 [1–3]	2 [2–3]	0.610
Labor phase, *n* (%)	Latent: 27 (65.85) Active: 14 (34.15)	Latent: 21 (55.26) Active: 17 (44.74)	0.3355
Oxytocin administered, *n* (%)	Yes: 19 (46.34)	Yes: 24 (63.16)	0.1337
Oxytocin dose (IU)	0 [0–5]	5 [0–5]	0.181
Parity at labour admission (number of previous births)	2 [1–3]	1.5 [1–2]	0.127
Epidural block, *n* (%)	41 (100)	38 (100)	NA

*Note*: Values are presented as median [Q1–Q3] for continuous variables and *n* (%) for categorical variables. *P*‐values are from two‐sided Mann–Whitney *U*‐test (continuous variables) or chi‐square test (categorical variables). Values shown in bold indicate statistical significance. Parity was defined as the number of previous births at the time of labour admission; the current delivery was not included. Abbreviations: BMI, body mass index; NA, not applicable.

### 3.2 Dynamics of cardiac autonomic indices and uterine activity irregularity

**FIGURE 3:**
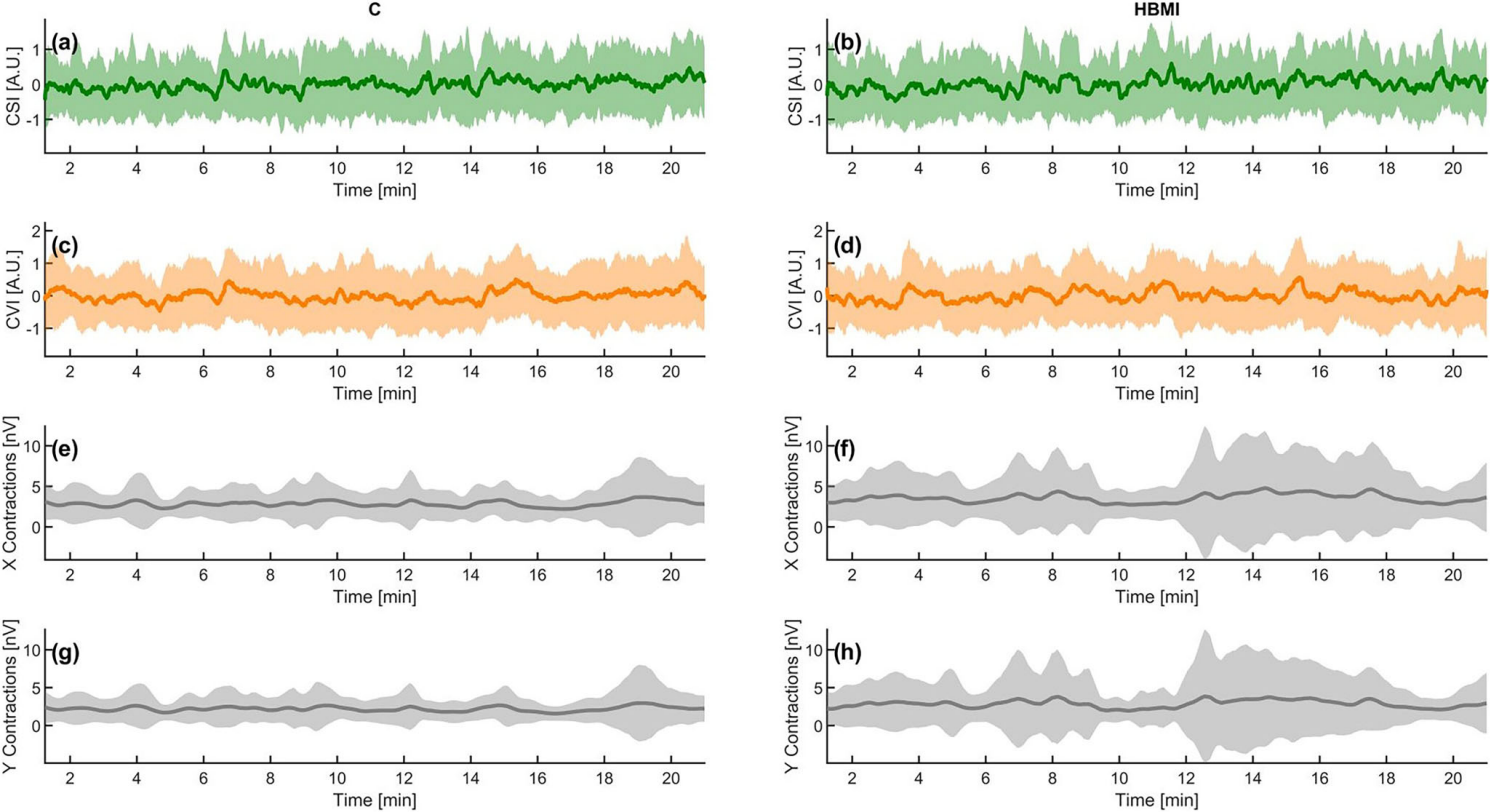
Physiological time series in normal‐weight and high BMI groups. All panels display physiological time series for the 20‐min segment analysed. The first row (a, b) shows the cardiac sympathetic index (CSI), and the second row (c, d) depicts the cardiac vagal/parasympathetic index (CVI). The third row (e, f) corresponds to TOCO‐like uterine activity derived from the horizontal or *X* component, while the fourth row (g, h) represents the vertical or *Y* component. Columns correspond to normal‐weight/Control (C) and high BMI (HBMI) groups, respectively. Bold continuous lines represent the group‐level mean values, and shaded bands indicate inter‐subject variability. Units are expressed as arbitrary units (A.U.) for CSI and CVI, and nanovolts (nV) for uterine activity.

Figure 3 presents the 20‐min group‐level trajectories of the cardiac autonomic indices (CSI, green; CVI, orange) and the TOCO‐like uterine activity envelopes (grey) along the *X*‐ and *Y*‐components. CSI traces (Figure 3a,b) and CVI traces (Figure 3c,d) show visually higher mean levels and broader fluctuations in the HBMI group (Figure 3b,d), with the normal‐weight/Control group displaying lower values (Figure 3a,c). Similarly, uterine activity along the *X*‐component (Figure 3e,f) and *Y*‐component (Figure 3 g,h) appears visually more regular in the Control group (Figure 3e,g), while the high BMI group (Figure 3f,h) exhibits larger and less regularly timed bursts.

**FIGURE 4:**
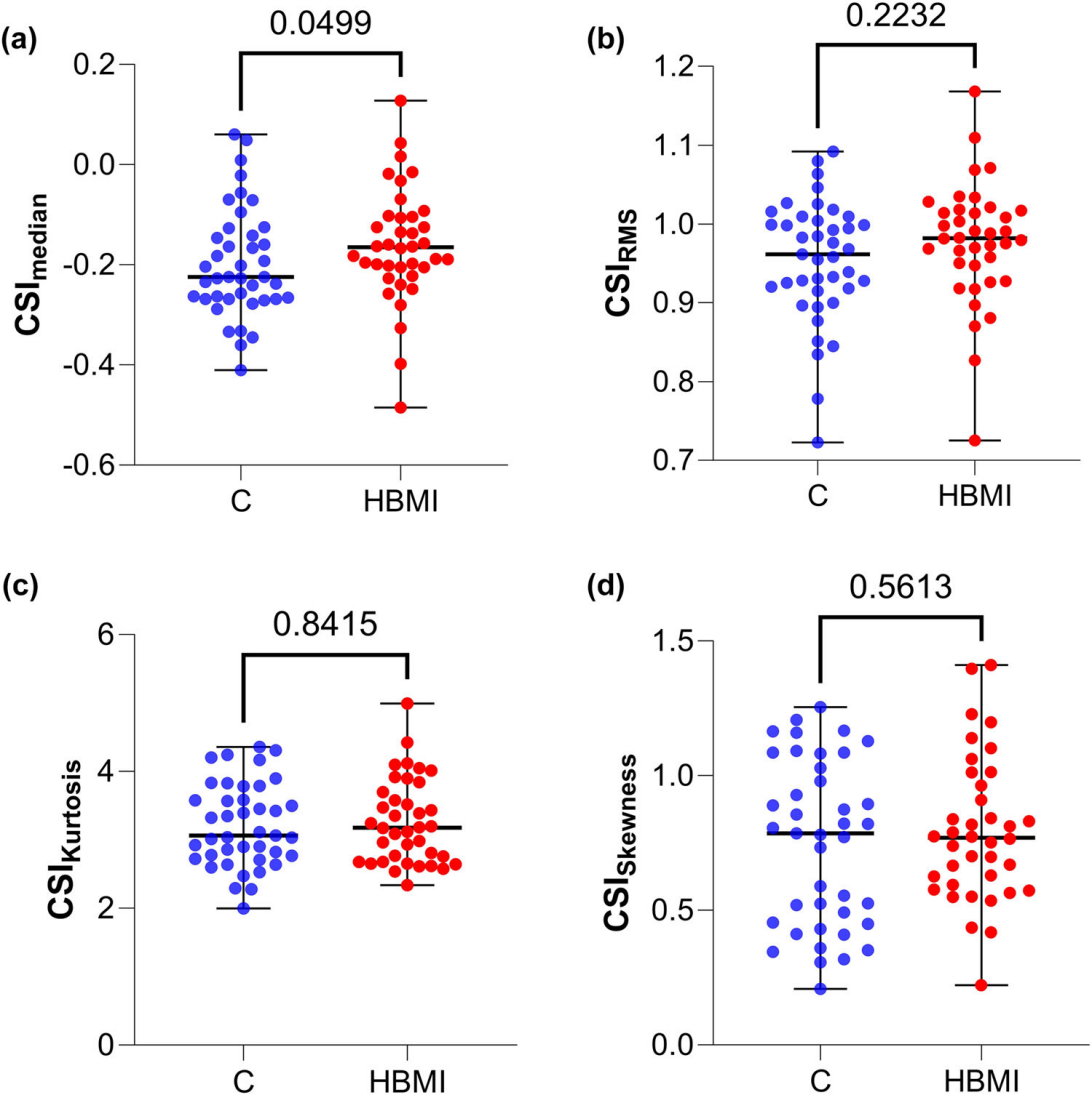
Statistical characteristics of the cardiac sympathetic index (CSI) by group. Panels show the participant‐level CSI median (a), RMS value (b), kurtosis (c), and skewness (d) for Control (C, blue) and high BMI (HBMI, red). Each dot represents the participant‐level median across 5‐min windows (10‐s step); group data are median with range. Between‐group comparisons were performed using the Mann–Whitney *U*‐test (two‐sided, α = 0.05); *P*‐values are shown above brackets.

In Figure 4a, the participant‐level CSI_median_ is higher (i.e., less negative) in HBMI than in C (*P* = 0.0499; C: −0.224 [−0.268 to −0.135] vs. HBMI: −0.165 [−0.209 to −0.104]). Physiologically, higher CSI values are commonly interpreted as reflecting a relative shift toward greater cardiac sympathetic modulation. In contrast, Figure 4b–d shows no between‐group differences for CSI_RMS_ (*P* = 0.2232), CSI_kurtosis_ (*P* = 0.8415) or CSI_skewness_ (*P* = 0.5613), suggesting broadly comparable dispersion and distributional shape of CSI fluctuations across groups.

**FIGURE 5:**
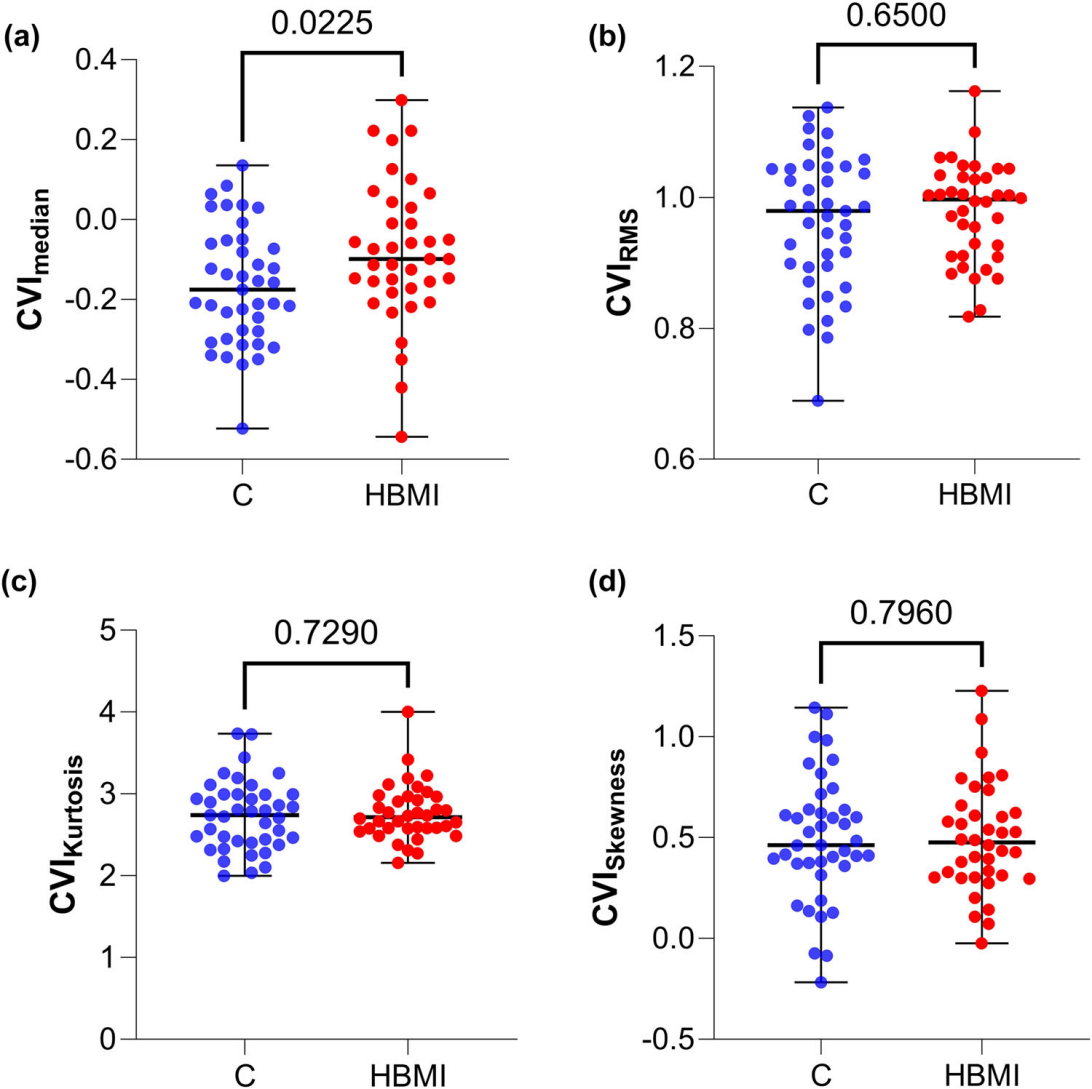
Statistical characteristics of the cardiac vagal/parasympathetic index (CVI) by group. Panels show the participant‐level CVI median (a), RMS value (b), kurtosis (c), and skewness (d) for Control (C, blue) and high BMI (HBMI, red). Each dot represents the participant‐level median across 5‐min windows (10‐s step); group data are median with range. Between‐group comparisons were performed using the Mann–Whitney *U*‐test (two‐sided, *α* = 0.05); *P*‐values are shown above brackets.

A similar pattern is observed for CVI. In Figure 5a, the participant‐level CVI_median_ is higher in HBMI than in C (*P* = 0.0225; C: −0.176 [−0.289 to −0.056] vs. HBMI: −0.099 [−0.175 to 0.033]). Because CVI is typically viewed as an index of cardiac vagal/parasympathetic modulation, higher CVI values are consistent with a relative shift toward greater vagal influence. By comparison, Figure 5b–d shows no significant between‐group differences for CVI_RMS_ (*P* = 0.6500), CVI_kurtosis_ (*P* = 0.7290) or CVI_skewness_ (*P* = 0.7960), indicating similar variability and distributional characteristics across groups.

**FIGURE 6:**
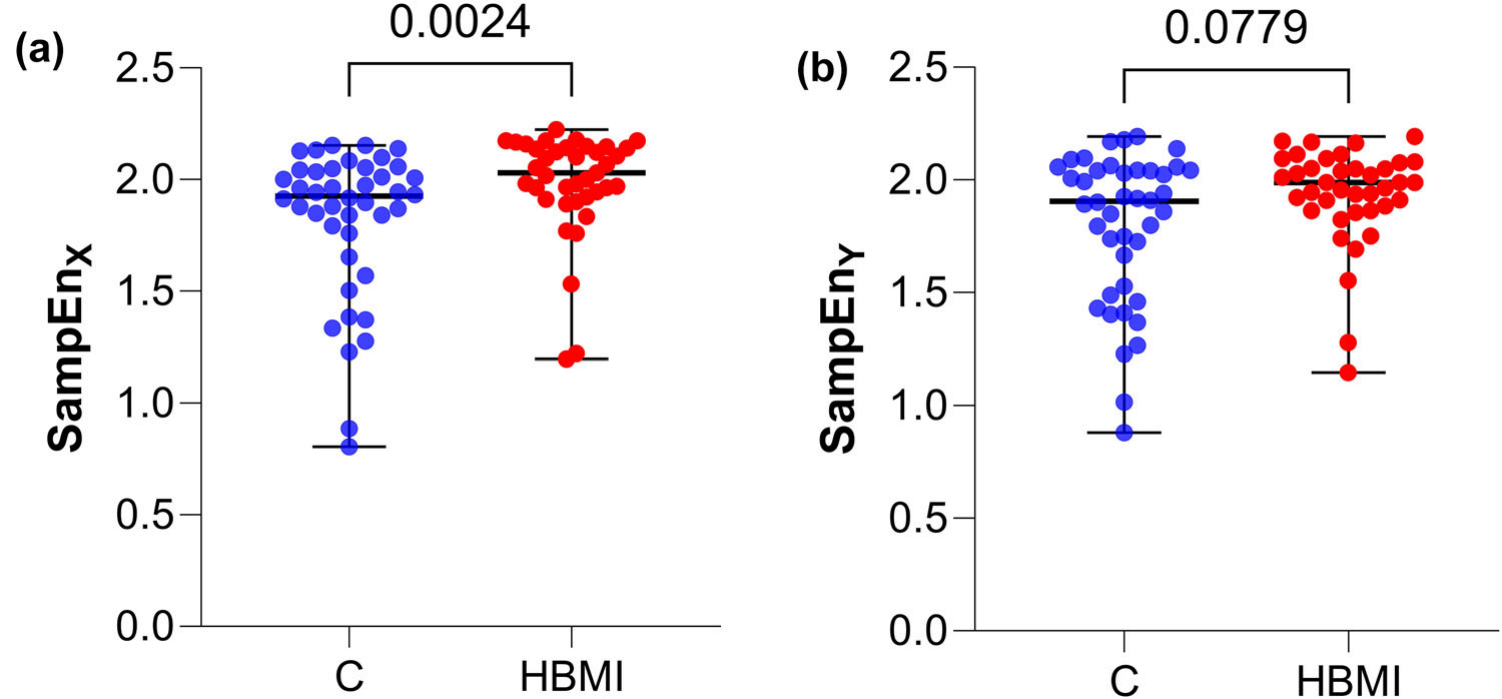
Sample entropy of TOCO‐like uterine activity by BMI group. Panels show sample entropy (SampEn) for the *X* (a) and *Y* (b) components in Control (C, blue) and high BMI (HBMI, red). SampEn values are shown as individual participants (dots), with group data displayed as the median and range. Between‐group comparisons were performed using the Mann–Whitney *U*‐test (two‐sided, α = 0.05); *P*‐values are reported above brackets.

Regarding uterine activity irregularity, Figure 6a shows that SampEn of the TOCO‐like signal is higher in HBMI than in C along the *X* component (*P* = 0.0024; C: 1.925 [1.733–2.044] vs. HBMI: 2.029 [1.922–2.142]). Higher SampEn values generally indicate greater irregularity of the uterine activity time series. In Figure 6b, the *Y* component does not differ significantly between groups (*P* = 0.0779), suggesting that this irregularity shift is more evident along the *X* direction in these recordings.

As a complementary, alignment‐free analysis, we assessed within‐group associations between participant‐level CSI/CVI and the mean envelope amplitude of the TOCO‐like uterine activity (Supporting information, Figures S1 and S2); these correlations were not significant, suggesting that uterine envelope amplitude (a proxy of contraction intensity in the EHG‐derived TOCO‐like signal) does not by itself explain inter‐individual variability in autonomic indices.

### 3.3 Time‐resolved phase‐locking and mean phase difference between autonomic indices and uterine contractions

**FIGURE 7:**
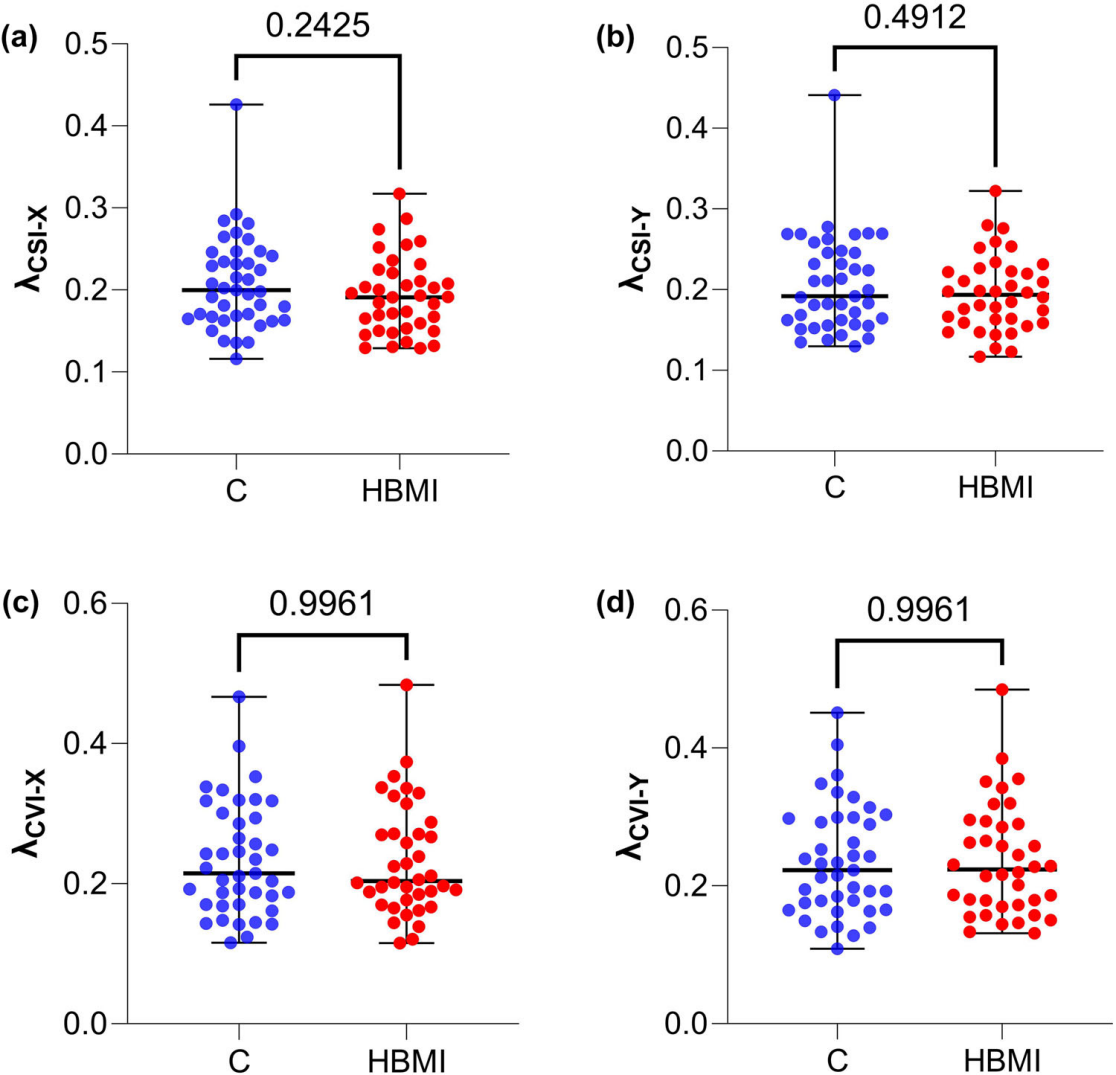
Phase synchronization between autonomic indices and TOCO‐like uterine activity by BMI group. Panels show the phase‐synchronization index (λ) between CSI and TOCO‐like uterine activity in the *X* (a) and *Y* (b) components, and between CVI and TOCO‐like activity in *X* (c) and *Y* (d), for Control (C, blue) and high BMI (HBMI, red). Each dot represents the participant‐level median across 5‐min windows (10‐s step); group data are median with range. Between‐group comparisons were performed using the Mann–Whitney *U*‐test (two‐sided, α = 0.05); *P*‐values are reported above brackets.

**FIGURE 8:**
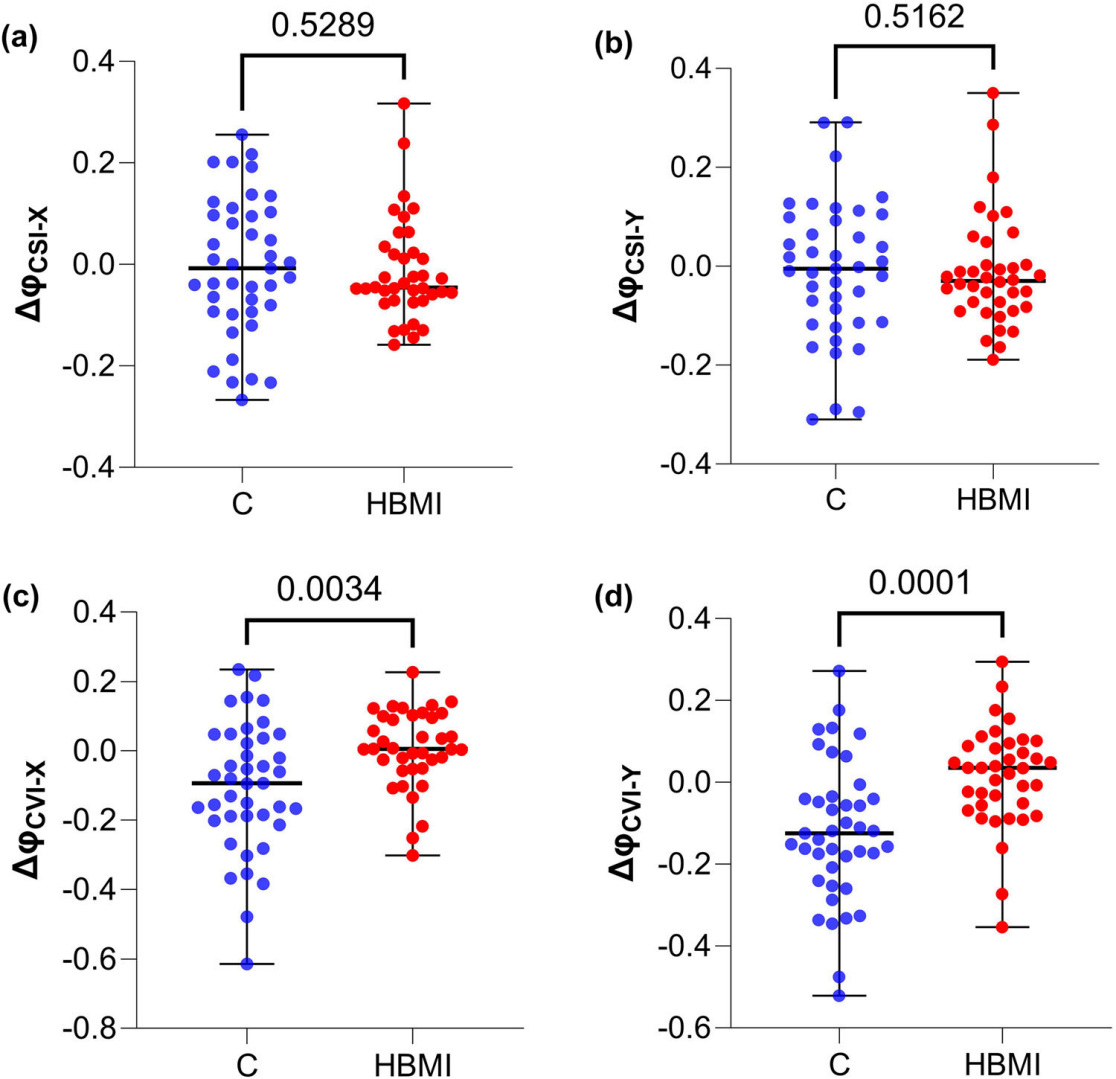
Mean phase difference between autonomic indices and TOCO‐like uterine activity by BMI group. Panels show the mean phase difference (Δφ, in units of π radians) between CSI and TOCO‐like uterine activity in the *X* (a) and *Y* (b) components, and between CVI and TOCO‐like activity in *X* (c) and *Y* (d), for Control (C, blue) and high BMI (HBMI, red). Each dot represents the participant‐level median across 5‐min windows (10‐s step); group data are median with range. Between‐group comparisons were performed using the Mann–Whitney *U*‐test (two‐sided, α = 0.05); *P*‐values are reported above brackets.

Turning to autonomic–uterine coupling, Figure 7a–d indicates that the λ does not differ between C and HBMI for any pairing (CSI–*X*: *P* = 0.2425; CSI–*Y*: *P* = 0.4912; CVI–*X*: *P* = 0.9961; CVI–*Y*: *P* = 0.9961). Since larger λ reflects stronger phase‐locking (tighter synchronization), these results suggest broadly similar overall phase synchronization strength between groups at the participant level. Finally, in Figure 8a,b, the mean phase difference (Δφ) likewise shows no group differences for CSI–TOCO pairs (CSI–*X*: *P* = 0.5289; CSI–*Y*: *P* = 0.5162). In contrast, Figure 8c,d reveals significant between‐group shifts in CVI‐related phase differences with HBMI values displaced toward/through zero relative to C (CVI–*X*: *P* = 0.0034; C: −0.093 [−0.195 to 0.042] vs. HBMI: 0.005 [−0.051 to 0.100]; CVI–*Y*: *P* = 0.0001; C: −0.124 [−0.224 to −0.037] vs. HBMI: 0.035 [−0.059 to 0.090]). These shifts indicate altered vagal–uterine phase timing (i.e., a systematic change in relative lead/lag and proximity to in‐phase timing under our convention), while overall λ remained comparable between groups.

We also analysed BMI as a continuous predictor of coupling (see Supporting information, Figures S3 and S4). There were no significant links between BMI and λ across any pairings (Figure S3). However, BMI showed positive correlations with Δφ for CVI–TOCO in both *X* and *Y* directions, suggesting an association between higher BMI and more positive Δφ values (a shift in vagal–uterine phase timing), while λ showed no significant associations.

## 4 DISCUSSION

Maternal autonomic dynamics and uterine activity during the first stage of labour presented differences between Control and high BMI groups. Overall, our participant‐level results suggest distributional shifts in autonomic indices and their timing relationships with uterine activity in HBMI participants. Uterine activity appeared more regular in controls, whereas HBMI individuals exhibited less predictable patterns—particularly along the horizontal component. Entropy indicated greater irregularity in HBMI individuals, and phase‐based analyses suggested a shift in vagal–uterine phase timing, specifically for CVI‐related phase differences, highlighting the potential importance of parasympathetic modulation in autonomic–uterine interactions during the first stage of labour.

The CSI and CVI offer time‐resolved, interpretable proxies of autonomic outflow, already validated in diverse contexts. They have distinguished autonomic responses to social versus self‐touch (Candia‐Rivera et al., 2025a), tracked pharmacologically induced sympathetic reactivity in hypertensive and control rats (Candia‐Rivera et al., 2026), and revealed bilateral maternal–fetal cardiac interactions that intensify during labour (Candia‐Rivera & Chavez, 2025). Complementarily, our group has demonstrated that uterine activity modulates both fetal and maternal heart rhythms: fetal cardio‐electrohysterographic coupling is already detectable in late pregnancy (Montero‐Nava et al., 2020), and maternal cardio‐electrohysterographic coupling or MCEC strengthens during labour (Esquivel‐Arizmendi et al., 2020). Collectively, these studies support CSI/CVI as robust tools to capture autonomic and physiological interactions across preclinical and clinical domains. In this cohort, excess adiposity was associated with changes at the participant level in autonomic indices: HBMI exhibited a higher median in CSI and CVI compared to controls, while measures of variability and shape (RMS, kurtosis, skewness) were generally similar across groups (Figures 4 and 5). These maternal‐side findings align with fetal HRV results from the same database. Two studies reported that, during labour, fetuses of obese mothers show patterns consistent with parasympathetic predominance and blunted sympathetic activation—higher RMSSD and frequency‐domain shifts, and symbolic‐dynamics changes—relative to fetuses of normal‐weight mothers (Martínez‐Reyna Emiliano and Dorantes‐Méndez, 2025; Martínez‐Reyna Emiliano and Reyes‐Lagos, 2025). This concordance might indicate a dyadic maternal–fetal autonomic environment that shifts towards vagal control when maternal BMI is high during labour.

A methodological novelty here is quantifying irregularity directly in TOCO‐like envelopes derived from rotated EHG components (horizontal *X*, vertical *Y*), rather than focusing solely on the raw EHG. We found higher sample entropy along *X* in HBMI than in controls (Figure 6a), whereas *Y* showed no group differences (Figure 6b). This axis‐specific increase in unpredictability may suggest that excess adiposity disrupts the timing and organization of contraction bursts preferentially along a predominant propagation direction, offering a signal closer to mechanical output and clinical interpretation (Reyes‐Lagos et al., 2024).

Since oxytocin influences both uterine contractions and autonomic regulation, the increased uterine irregularity seen in the HBMI group should not be solely attributed to BMI. Instead, these patterns might result from the combined effects of maternal body habitus and the intrapartum treatment context. In our cohort, oxytocin exposure was similar across groups; however, the exact timing and duration of administration relative to the physiological segments analysed were unavailable. Therefore, oxytocin‐related effects remain a plausible factor in the observed autonomic–uterine interactions.

In our phase‐based characterization, CSI–TOCO phase‐locking did not differ between C and HBMI across any autonomic–uterine pairing (Figure 7), whereas mean phase differences differed for CVI–TOCO in both *X* and *Y* (Figure 8c,d). Moreover, analysing BMI as a continuous variable showed no significant associations between BMI and λ (Supporting information, Figure S3), while BMI was positively associated with Δφ for CVI–TOCO in *X* and *Y* (Supporting information, Figure S4c,d), suggesting that higher BMI relates to more positive Δφ values (a systematic shift in vagal–uterine phase timing). Taken together, these findings suggest that excess adiposity may alter vagal–uterine timing relationships without necessarily changing overall phase‐locking strength.

Several mechanisms may reshape MCEC in the context of excess adiposity. Adipokines linked to obesity—particularly leptin and visfatin—directly inhibit human myometrial contractility, implying a blunted sympathetic effectiveness at the uterus; this aligns with established adrenergic pharmacology in which α‐adrenergic drive favours contraction and β‐adrenergic signalling favours relaxation (Liu et al., 1998; Moynihan et al., 2006; Mumtaz et al., 2015). Maternal obesity and high‐fat diet are also associated with impaired uteroplacental perfusion and placental dysfunction, a pathway that might disrupt cardio‐uterine timing (Frias et al., 2011; Howell & Powell, 2017; Louwen et al., 2024; Teulings et al., 2020). Beyond tissue‐level effects, both obesity and pregnancy reduce baroreflex sensitivity, and labour pain and analgesic interventions (when used) acutely modulate autonomic tone and fetal heart‐rate patterns—factors that reshape short‐term cardiovascular–uterine dynamics (Brooks et al., 2012; Konstantinidou et al., 2021; Riveros‐Perez et al., 2023). Thus, these pathways could plausibly shift phase timing and/or alter phase‐locking strength during labour, potentially altering the balance of autonomic influences, whereas vagal pathways may remain comparatively robust.

The primary limitation is the small sample size in the initial obesity subgroup, which led us to combine overweight and obesity into HBMI to enhance the reliability and interpretability of our findings. Although we recorded key potential confounders (labour phase, parity and oxytocin administration/dose) and found no significant between‐group differences (Table 1), we did not model these covariates in multivariable analyses given the limited sample size; thus, residual confounding cannot be excluded. Epidural block was not administered in either group. Future research should focus on: (i) analysing contraction‐locked and stage‐specific segments to better capture labour progression effects, (ii) integrating baroreflex surrogates and pain/analgesia status when applicable, (iii) combining directional EHG features with phase‐based coupling, and (iv) validating results with biochemical markers (e.g., catecholamines and adipokines) in larger, stratified cohorts.

Additionally, while oxytocin exposure did not show significant differences between groups, the precise timing and duration of oxytocin administration relative to the analysed segments were unavailable. As a result, it was not possible to assess how treatment might have modulated autonomic–uterine dynamics over time, and such effects cannot be ruled out.

### 4.1 Conclusion

Our study suggests that higher BMI is associated with differences in maternal autonomic control and its relationship with uterine activity during labour. The high BMI group showed higher median CSI and CVI values, consistent with overall autonomic overactivation (i.e., increased sympathetic and vagal‐related modulation) compared with normal‐weight parturients, as captured by these time‐resolved proxies. Uterine irregularity was more prominent in high BMI participants within the analysed labour segments, although part of this pattern may also reflect concurrent intrapartum management. Phase analysis further showed that vagal‐related mean phase differences (Δφ) were shifted in HBMI and positively correlated with BMI, suggesting a BMI‐related alteration of MCEC primarily expressed as altered vagal–uterine phase timing (i.e., a shift in lead/lag structure). Notably, phase‐locking strength (λ) was broadly comparable, suggesting that BMI‐related differences were expressed primarily as timing shifts rather than changes in overall synchronization strength. These results support the use of time‐resolved CSI/CVI indices with EHG as non‐invasive, mechanistically meaningful markers of autonomic–uterine interaction. Clinically, BMI‐sensitive monitoring could improve intrapartum risk assessment and support personalized management decisions.

## AUTHOR CONTRIBUTIONS

Carlos Gabriel Varela‐Albarrán: Conceptualization, data curation, methodology, software, formal analysis, visualization, writing—original draft. José Javier Reyes‐Lagos: Conceptualization, supervision, methodology, funding acquisition, project administration, writing—review and editing. Laura Mercedes Santiago‐Fuentes: Investigation, participant recruitment, data curation, writing—review and editing. Guadalupe Dorantes‐Méndez: Methodology, statistical analysis, validation, writing—review and editing. Eric Alonso Abarca‐Castro: Signal processing methodology, software validation, resources, writing—review and editing. Paula Romina Soria: Investigation, visualization, writing—review and editing. Araceli Espinosa‐Guerrero: Clinical supervision, resources, investigation, writing—review and editing. All authors gave final approval for publication and agree to be accountable for all aspects of the work. All authors have read and approved the final version of this manuscript and agree to be accountable for all aspects of the work in ensuring that questions related to the accuracy or integrity of any part of the work are appropriately investigated and resolved. All persons designated as authors qualify for authorship, and all those who qualify for authorship are listed.

## CONFLICT OF INTEREST

None declared.

## Supporting information



Figures S1–S4.

## Data Availability

Anonymized data supporting the findings (maternal RRI time series and EHG‐derived TOCO‐like envelopes) are deposited in the institutional repository of UAM Lerma (http://hdl.handle.net/20.500.12222/440; https://xogi.ler.uam.mx/items/dbc341cc‐97d0‐40d5‐952c‐79d48c3538fe). For the present analysis, the HBMI group combines participants from the Overweight and Obesity categories.
