# Supplementary material for: Higher body‐mass index modifies time‐resolved maternal autonomic cardiac–uterine coupling during the first stage of human labour
Source: Exp Physiol. 2026 Apr 25;111(7):3203–15. doi: 10.1113/EP093339 (PMC13327326; doi:10.1113/EP093339)
Supplement: Supplementary file 1 — Figures S1–S4. [file EPH-111-3203-s001.docx]

**Supplementary Material**

**Supplementary Figure S1 (Control group)**

**
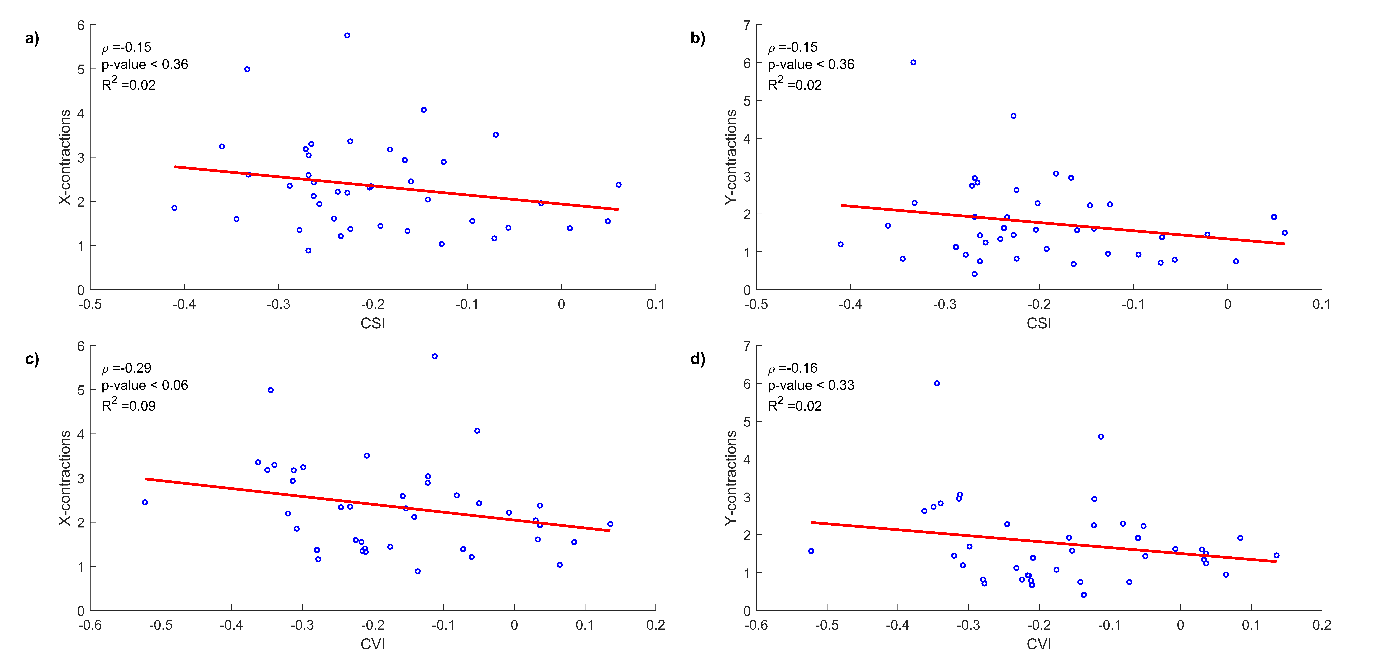
**

Supplementary Figure S1. Within-group association between autonomic indices and TOCO-like envelope amplitude (Control). Scatterplots show the relationship between participant-level CSI and the mean envelope amplitude of the TOCO-like uterine activity in the X component (a) and Y component (b), and between participant-level CVI and the mean envelope amplitude in X (c) and Y (d), within the Control group. Each dot represents one participant. The red line indicates the linear fit. Spearman’s rank correlation (ρ) and its p-value are reported in each panel; R² corresponds to the displayed linear fit.

**Supplementary Figure S2 (HBMI group)**


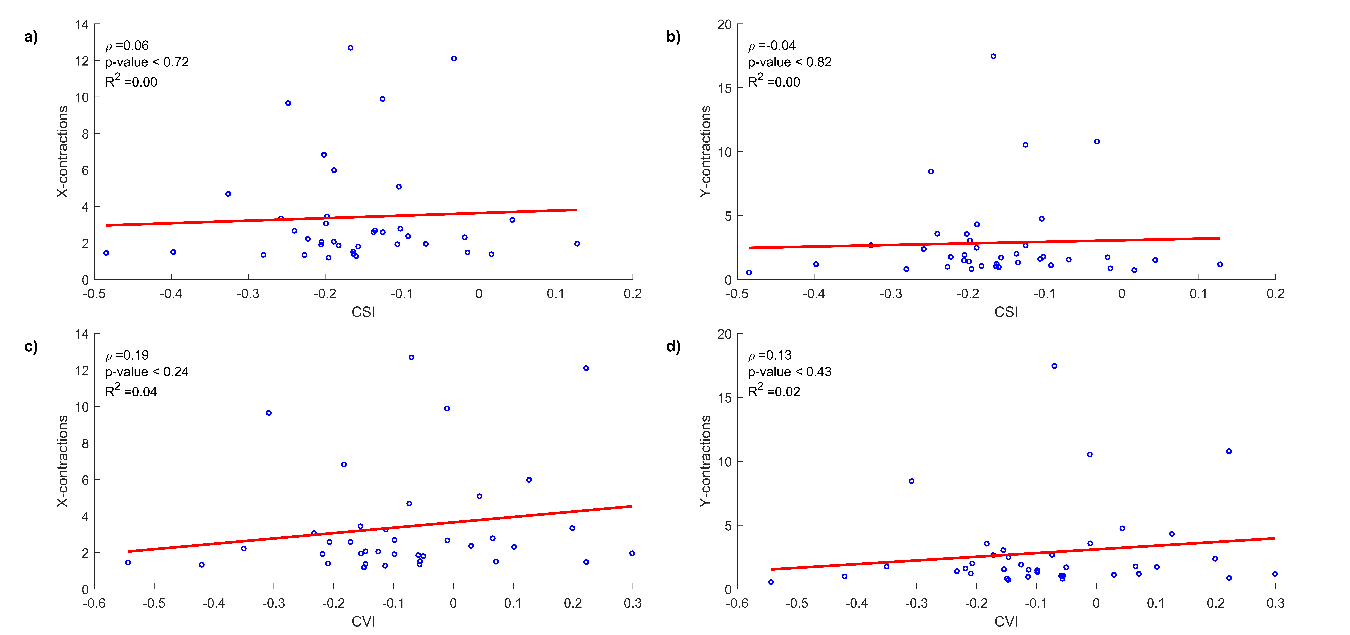


Supplementary Figure S2. Within-group association between autonomic indices and TOCO-like envelope amplitude (High BMI or HBMI). Scatterplots show the relationship between participant-level CSI and the mean envelope amplitude of the TOCO-like uterine activity in the X component (a) and Y component (b), and between participant-level CVI and the mean envelope amplitude in X (c) and Y (d), within the HBIM group. Each dot represents one participant. The red line indicates the linear fit. Spearman’s ρ, p-values, and R² for the displayed fit are reported in each panel.

**Supplementary Figure S3 (All participants)**


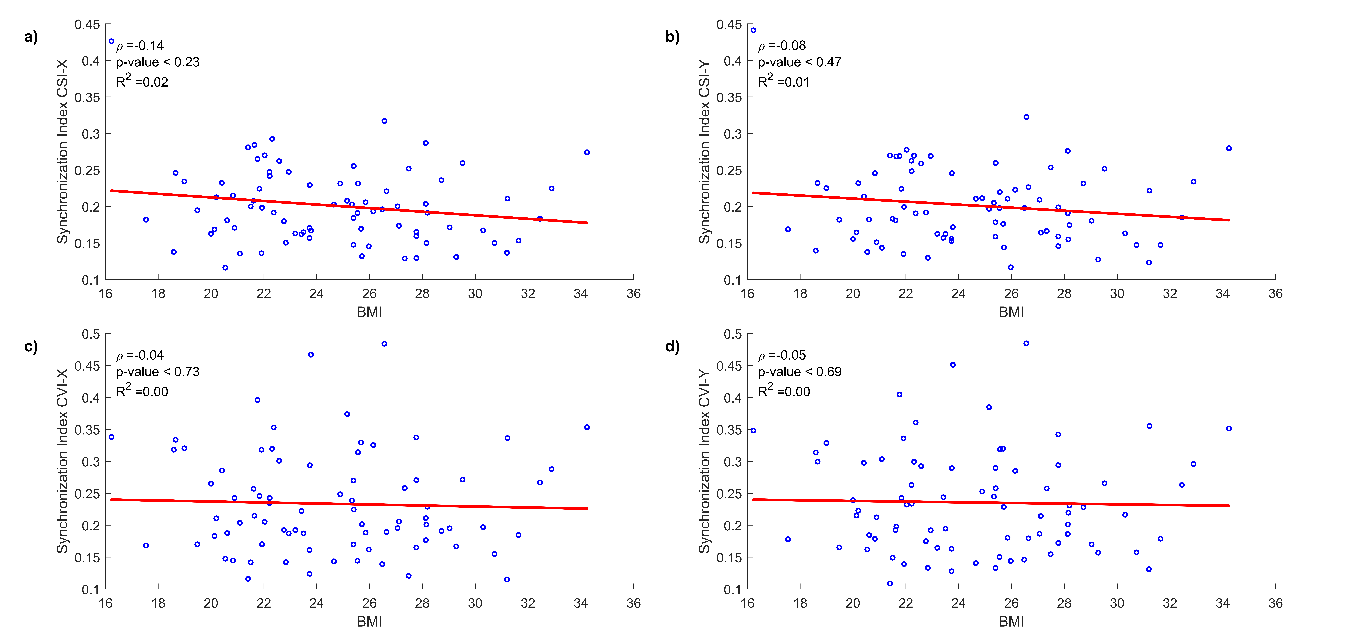


Supplementary Figure S3. Relationship between BMI and phase synchronization (all participants). Scatterplots show the association between BMI and the phase-synchronization index λ between CSI and TOCO-like uterine activity in X (a) and Y (b), and between CVI and TOCO-like activity in X (c) and Y (d), pooling all participants. Each dot represents one participant. The red line indicates the linear fit. Spearman’s ρ, p-values, and R² are reported in each panel.

**Supplementary Figure S4 (All participants)**

**
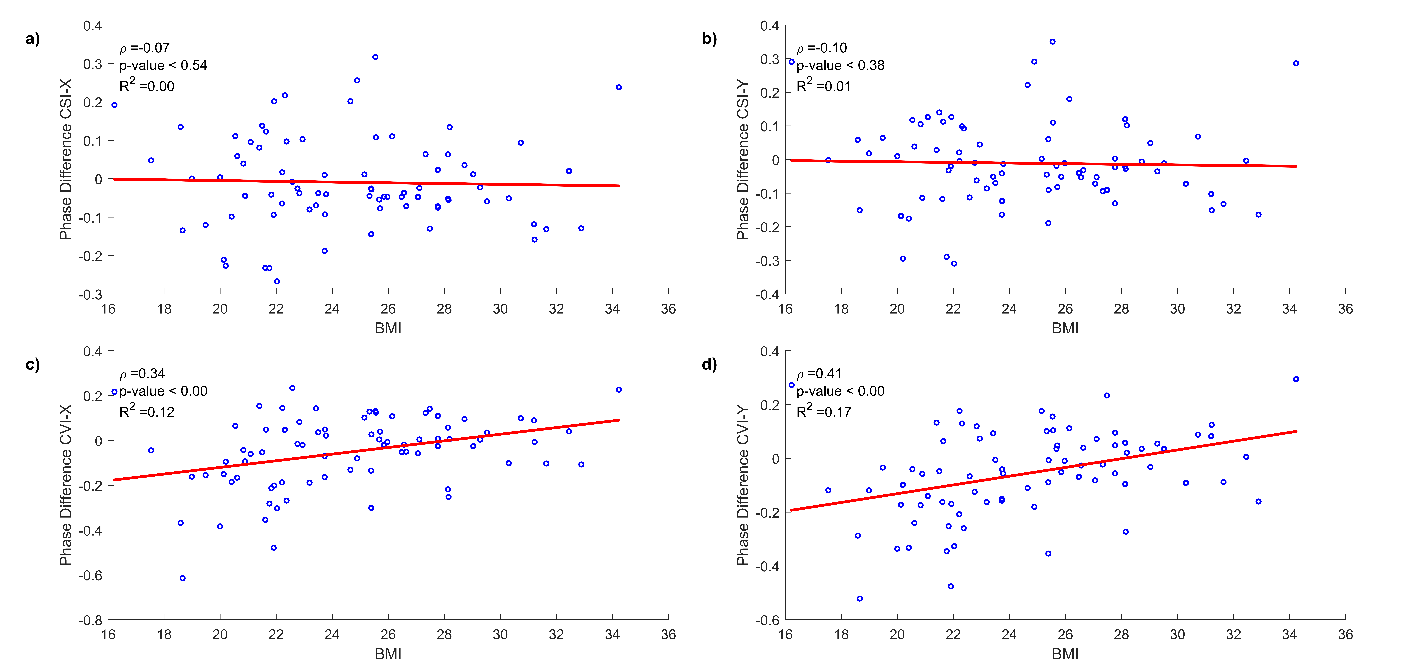
**

Supplementary Figure S4. Relationship between BMI and mean phase difference (all participants). Scatterplots show the association between BMI and the mean phase difference Δφ between CSI and TOCO-like uterine activity in X (a) and Y (b), and between CVI and TOCO-like activity in X (c) and Y (d), pooling all participants. Each dot represents one participant. The red line indicates the linear fit. Spearman’s ρ, p-values, and R² are reported in each panel.
